# Portable magnetic resonance imaging of patients indoors, outdoors and at home

**DOI:** 10.1038/s41598-022-17472-w

**Published:** 2022-07-30

**Authors:** Teresa Guallart-Naval, José M. Algarín, Rubén Pellicer-Guridi, Fernando Galve, Yolanda Vives-Gilabert, Rubén Bosch, Eduardo Pallás, José M. González, Juan P. Rigla, Pablo Martínez, Francisco J. Lloris, Jose Borreguero, Álvaro Marcos-Perucho, Vlad Negnevitsky, Luis Martí-Bonmatí, Alfonso Ríos, José M. Benlloch, Joseba Alonso

**Affiliations:** 1 Tesoro Imaging S.L., 46022 Valencia, Spain; 2 Institute for Molecular Imaging and Instrumentation, Spanish National Research Council, 46022 Valencia, Spain; 3 Institute for Molecular Imaging and Instrumentation, Universitat Politècnica de València, 46022 Valencia, Spain; 4 PhysioMRI Tech S.L., 46022 Valencia, Spain; 5 Asociación de investigación MPC, 20018 San Sebastián, Spain; 6 Intelligent Data Analysis Laboratory, Department of Electronic Engineering, Universitat de València, 46100 Burjassot, Spain; 7 Helios School, 46183 L’Eliana, Spain; 8 Oxford Ionics, Oxford, OX5 1PF UK; 9 Medical Imaging Department, Hospital Universitari i Politècnic La Fe, 46026 Valencia, Spain

**Keywords:** Magnetic resonance imaging, Imaging techniques

## Abstract

Mobile medical imaging devices are invaluable for clinical diagnostic purposes both in and outside healthcare institutions. Among the various imaging modalities, only a few are readily portable. Magnetic resonance imaging (MRI), the gold standard for numerous healthcare conditions, does not traditionally belong to this group. Recently, low-field MRI technology companies have demonstrated the first decisive steps towards portability within medical facilities and vehicles. However, these scanners’ weight and dimensions are incompatible with more demanding use cases such as in remote and developing regions, sports facilities and events, medical and military camps, or home healthcare. Here we present in vivo images taken with a light, small footprint, low-field extremity MRI scanner outside the controlled environment provided by medical facilities. To demonstrate the true portability of the system and benchmark its performance in various relevant scenarios, we have acquired images of a volunteer’s knee in: (i) an MRI physics laboratory; (ii) an office room; (iii) outside a campus building, connected to a nearby power outlet; (iv) in open air, powered from a small fuel-based generator; and (v) at the volunteer’s home. All images have been acquired within clinically viable times, and signal-to-noise ratios and tissue contrast suffice for 2D and 3D reconstructions with diagnostic value. Furthermore, the volunteer carries a fixation metallic implant screwed to the femur, which leads to strong artifacts in standard clinical systems but appears sharp in our low-field acquisitions. Altogether, this work opens a path towards highly accessible MRI under circumstances previously unrealistic.

## Introduction

Standard clinical MRI scanners make use of powerful superconducting magnets that interact strongly with the vast amount of hydrogen nuclei in the human body^1^. These magnets enable the high SNR and spatial resolution typical for magnetic resonance images. Regrettably, these magnets also require cryogenic refrigeration, they are bulky, heavy, expensive to build, site, operate and maintain, and they ultimately constitute a formidable barrier to the accessibility and democratization of MRI^2–4^. Besides, high-field scanners are subject to patient safety risks, e.g. due to projectile incidents^5^; they are limited in the imaging pulse sequences that can be played out due to increased specific absorption rates (SAR) of electromagnetic energy in tissues at the corresponding higher excitation radio-frequencies (RF)^6^; they generate undesirable acoustic noise due to strong magnetic interactions during scans^7^; and they induce severe image artifacts around metallic implants due to magnetic susceptibility effects^8–10^. Low-field systems ($<0.3$ T) can overcome all of the above and are nowadays gaining traction as affordable complements to standard MRI scanners. Recent achievements with low-field scanners include in vivo brain and extremity imaging^11,12^, hard-tissue imaging^13–15^ and even quantitative MRI and fingerprinting^16,17^. The main penalty to pay for operating in this regime is a significant loss in SNR and spatial resolution. However, the diagnostic value of the resulting reconstructions is not necessarily compromised, due to a number of reasons: (i) contrast-to-noise ratio (CNR), a more relevant metric for diagnosis than SNR, does not depend as strongly on field strength for some relevant contrast mechanisms^18,19^; (ii) multiple health conditions and diseases may be diagnosed without the exquisite detail provided by high-field images^2^; (iii) SAR constraints are less pronounced at low fields, allowing for efficient pulse sequences which increase the duty cycle to partly compensate the SNR loss^2^; and (iv) machine learning algorithms can be trained to recover image quality from noise-corrupted low-field data by e.g. transfer learning^20,21^.

The scope of conceivable applications for MRI technologies widens extraordinarily once the need for large superconducting magnets is removed. For instance, vehicles have been equipped with low-field systems^22,23^, and point-of-care and bedside neuroimaging have been demonstrated with a 64 mT FDA-cleared scanner^24,25^. The latter is arguably the most successful attempt at mobile MRI so far. However, it is based on a yoked magnet, which makes it heavy ($>600$ kg) and too large for standard door clear opening in residential constructions (32” in USA, 80 cm in Europe). Low-cost devices with improved mobility would enable MRI applications beyond clinical environments to home and hospice care, small clinics, rural areas or sports clubs and school facilities. Autonomously powered scanners could even be operated outdoors, e.g. in sports events, field hospitals or NGO and military camps^26^, making MRI available to a large fraction of the world population with no or insufficient access^2–4^.

In this article we present a 72 mT extremity MRI scanner based on a yokeless Halbach magnet mounted on a wheeled structure of width 70 cm, with an overall weight $\approx 250$ kg and component cost $<50$ k€. After checking that the system performs as expected for in vivo images under controlled ambient conditions in an MRI physics laboratory, we took images of the right knee of a volunteer in different indoor and outdoor environments, including the living room at the volunteer’s apartment, and in open air connected to a portable gasoline generator. All knee images were acquired with identical 3-dimensional Rapid Acquisition with Relaxation Enhancement (3D-RARE) sequences, in about 12 min each. The electromagnetic interference (EMI) spectrum was different at the various locations, which results in slightly different noise patterns in the reconstructed images. Nevertheless, they all yield valuable anatomical information in clinically acceptable times. The volunteer had undergone a femoral shaft osteotomy and carried a fixation metallic implant screwed to the femur. This hardware is sharply defined in our low-field acquisitions, where previous high-field images suffered from strong susceptibility-induced image distortions.

## Results

### Scanner performance in laboratory

**Figure 1:**
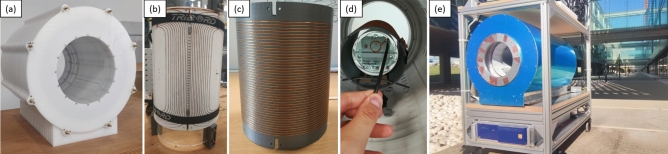
Photographs of the low-field extremity scanner: (**a**) 72 mT Halbach magnet; (**b**) gradient assembly; (**c**) RF Tx/Rx coil; (**d**) view of the scanner inside with a phantom in place; and (**e**) full system mounted on a transportable structure and in open air.

The system (Fig. 1) is built around a permanent magnet array in a Halbach configuration^12^, for a field strength of $\approx 72$ mT. Technical details on the apparatus can be found in the Methods section. The scanner is usually in the controlled environment of an MRI physics laboratory, where the temperature is stabilized at $18.0\pm 0.2$ C and the air relative humidity at $45\pm 10$%. In these conditions, the Larmor frequency is stable down to the kilo-hertz at 3.076 MHz over weeks, and minor corrections to the frequency tuning and impedance matching electronics suffice to compensate for the different electronic loading of the RF coil by different subjects and body parts. The abundant surrounding electronic equipment and scanners generate substantial EMI in the laboratory at frequencies within our detection bandwidth. For this reason, we conceal the resonant RF coil behind three grounded shields: one is the outermost scanner housing (blue in Fig. 1e), a 1.5 mm-thick copper sheet; another is inside the magnet between the RF coil and the gradient assembly, consisting of a series of 0.1 mm-thick copper tape strips of width 5 cm and tin-soldered along the seams for electrical continuity; and last is an electrically conducting cloth (Holland Shielding Systems, Dordrecht, the Netherlands) that can be wrapped around the subject at both scanner ends, to avoid antenna effects that otherwise couple EMI to the coil from the inside, despite the other two shields.

**Figure 2:**
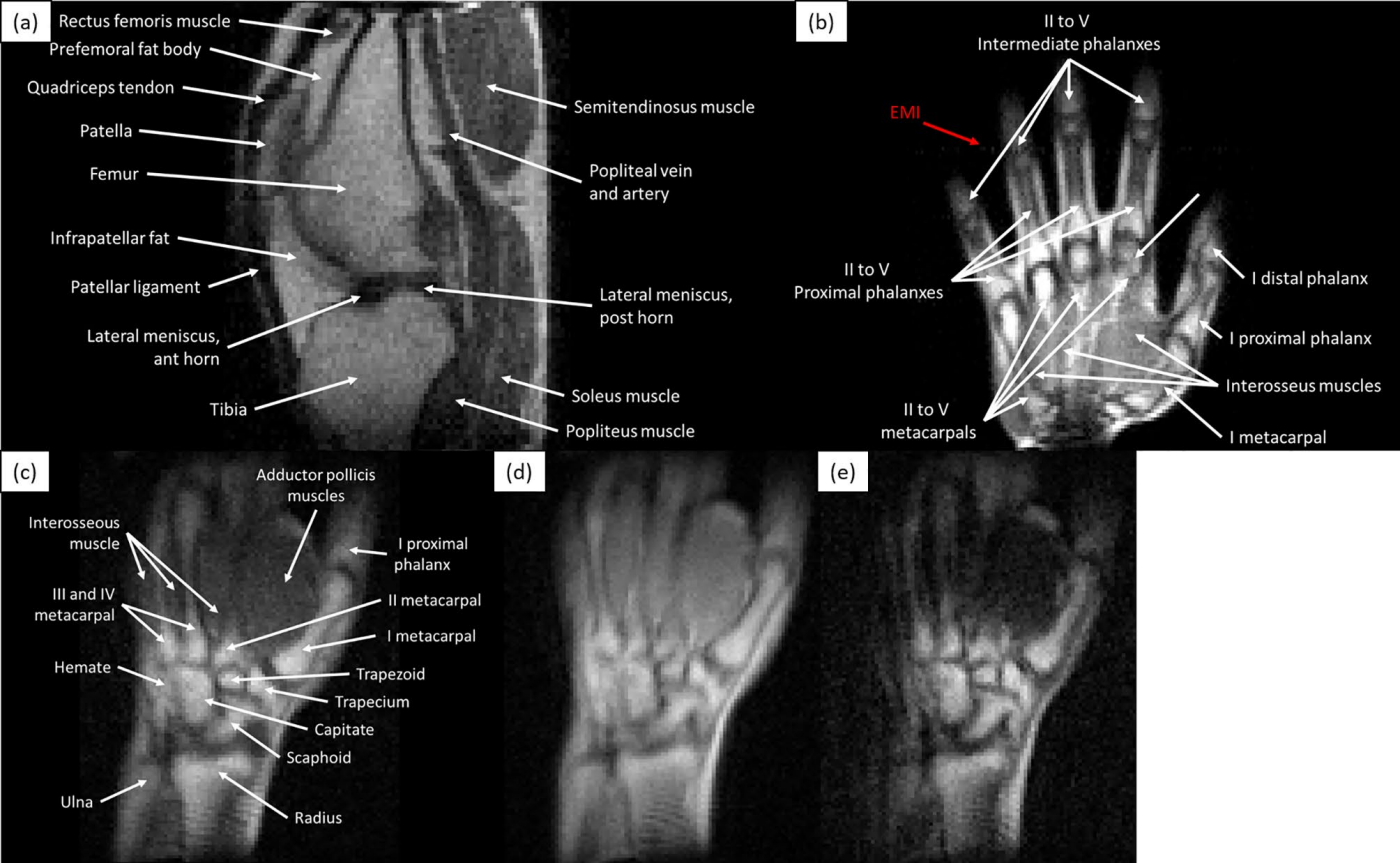
Single slices of 3D-RARE in vivo acquisitions of different volunteers in the MRI physics laboratory: (**a**) $T_1$-weighted image of a knee, acquired in 19 min; (**b**) $T_1$-weighted image of a hand (10 min), with a faint EMI line visible along the phase-encoded direction; (**c**)–(**e**) $T_1$, $\rho$ and $T_2$-weighted images of a wrist (12 min).

**Figure 3:**
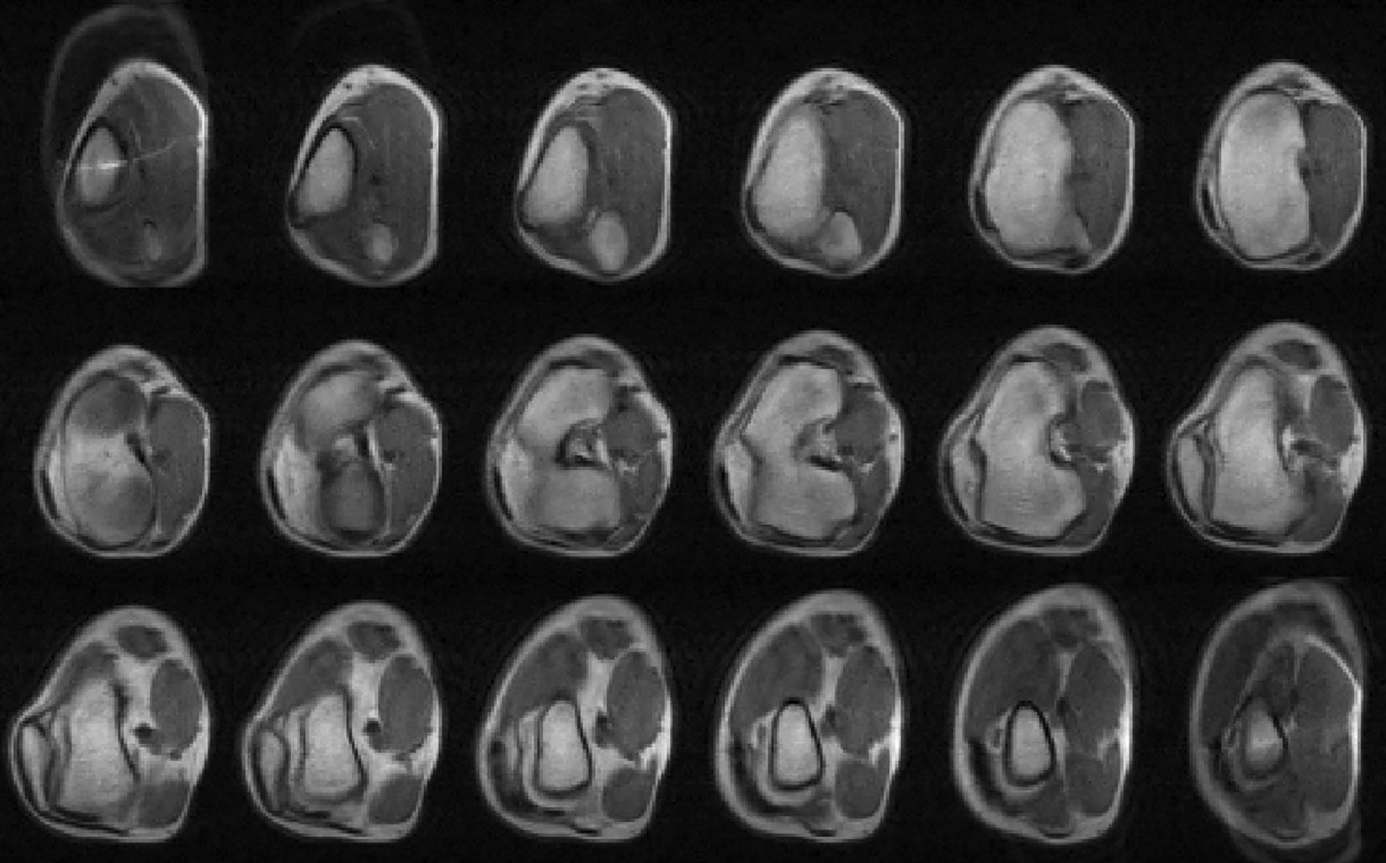
Complete set of axial slices of a $T_1$-weighted 3D-RARE knee acquisition (11.5 min), showing small distortions towards the edges of the field of view, and some aliasing between the first (top left) and last (bottom right) images.

The images in Figs. 2 and 3 show the scanner performance in the laboratory and correspond to in vivo 3D-RARE acquisitions of different healthy subjects on different days. The images in Fig. 2 show selected slices of a left knee, a right hand and a right wrist, with acquisition times ranging from 10 to 19 min (Methods). All images show sufficient tissue contrast and spatial resolution to identify relevant anatomical features, including muscles, fat, cortical bone, bone marrow, tendons, ligaments, veins, arteries and fascia. In these images we show different contrast mechanisms^1^, with weightings on $T_1$, $T_2$ and proton density ($\rho$). The images are unprocessed after Fourier reconstruction and weak EMI effects result in a faint line along the horizontal (phase-encoded) direction in Fig. 2b. Figure 3 includes the complete set of slices of an axial knee acquisition, showing small distortions towards the edges of the field of view due to non-ideal field distributions.

### In vivo imaging of metallic implants

**Figure 4:**
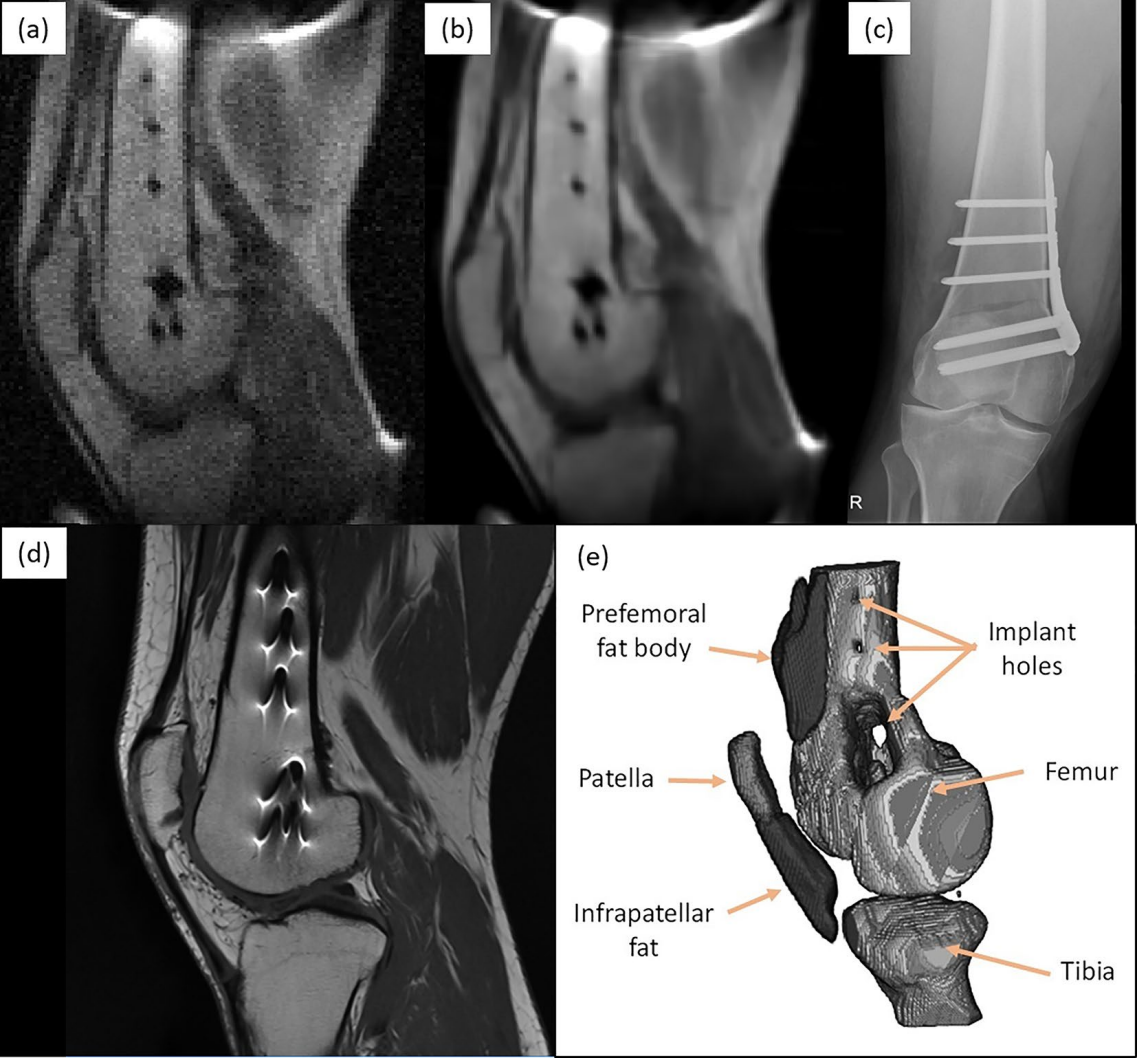
Images of fixation metallic implant attached to the femur, consisting of a plate and seven screws: (**a**) sagittal view of a raw low-field image acquired with the 72 mT system (9 mm slice from $T_1$-weighted 3D-RARE acquisition with in-plane resolution of $1.3\times 2$ mm$^2$, 12 min scan time, eight years after femoral shaft osteotomy); (**b**) same, but BM4D-filtered^27^ and rescaled by $\times 2$ to increase the number of pixels; (**c**) lateral X-ray computed radiography (two weeks after surgery); (**d**) sagittal view of the same knee, acquired with a Siemens Skyra 3 T system ($T_1$-weighted 2D-RARE acquisition with slice thickness 3.9 mm and pixel resolution $0.26\times 0.26$ mm$^2$, one year after surgery); and (**e**) 3D reconstruction from $T_1$-weighted 3D-RARE acquisition with isotropic resolution of 2 mm, 20 min scan time, where selected muscle and fat segments have been removed (eight years after surgery).

In a second set of experiments, we demonstrate in vivo MR images in the presence of metallic implants without the strong susceptibility-induced artifacts typical of high-field acquisitions^8,10^, which often hamper post-operative assessment of orthopedic procedures^9^. The volunteer for these tests had been diagnosed with lateral gonarthrosis due to cartilage damage in their right knee and had a femoral shaft osteotomy to remove pressure from the damaged tissue. The fixation metallic implant screwed to the femur is cleanly visible in a lateral X-ray computed radiographic image (Fig. 4c), but leads to high intensity fringes around the metallic hardware in high-field MR images due to incorrect spin mapping (see Fig. 4d, taken at 3 T). These effects depend supralinearly on the magnetic field strength and are barely perceptible at fields $<0.1$ T^28^. The field dependence is notorious in the images: the SNR and resolution are much higher in the 3 T system, but the metallic implant geometry is accurately defined in our 72 mT 2D and 3D reconstructions, and can be readily segmented with standard data post-processing. The low-field images were taken in 12 min (Fig. 4a,b) and 20 min (Fig. 4e) with $T_1$-weighted 3D-RARE acquisitions (Methods).

### Indoor, outdoor and residential MRI

**Figure 5:**
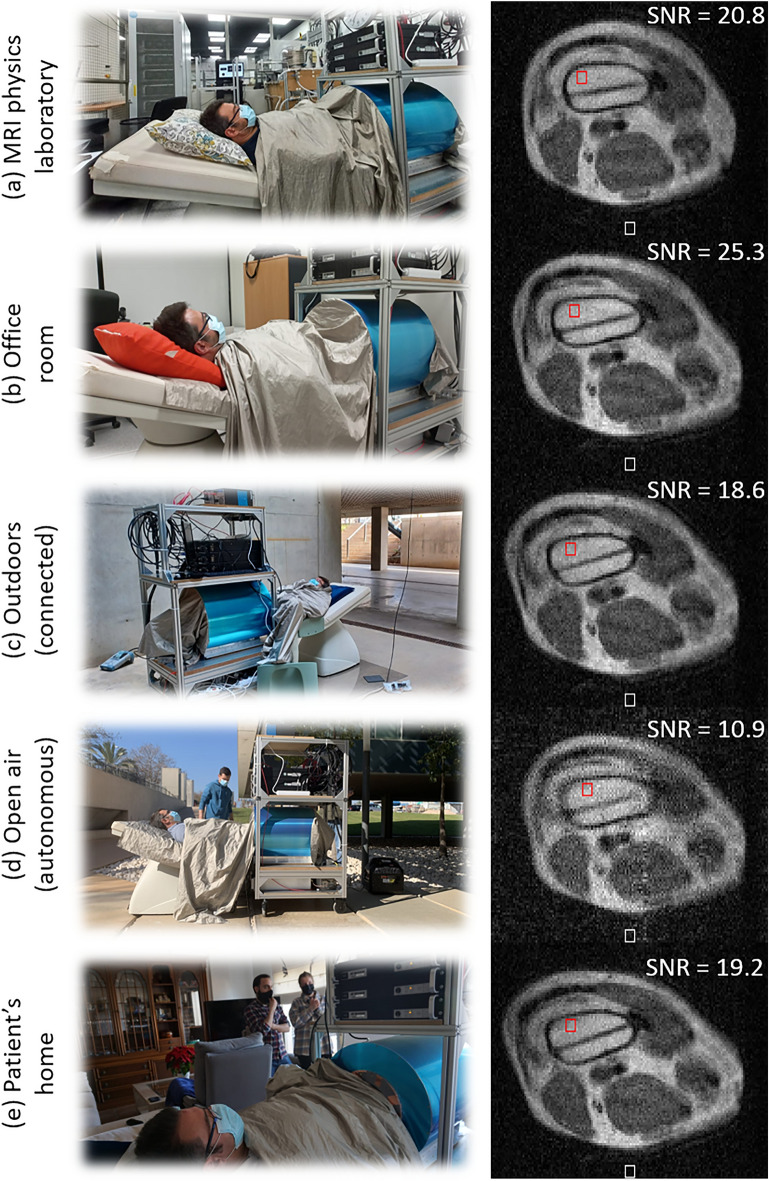
Photographs during acquisitions (left) and axial slice from 3D-RARE reconstructions (right, no post-processing) at five different locations: (**a**) in an MRI physics laboratory; (**b**) in an office room; (**c**) outside a campus building, connected to a nearby power outlet; (**d**) in open air, powered from a small fuel-based generator; and (**e**) at the volunteer’s home.

The goal of the last set of experiments is to evaluate the portability of the scanner and its performance under various environments and conditions. To establish comparisons as unbiased as possible, the acquisitions across all five scenarios are of the intervened knee of the same volunteer as in Fig. 4, and all with the same sequence parameters: $T_1$-weighted 3D-RARE with a total scan time $\approx 12$ min (Methods). The slices in Fig. 4 have been selected to show the third screw from the top (Fig. 4) as it runs through the bone from the top of the image, where the prosthetic plate is implanted. As a general indicator of the image quality, we measure the SNR in a region of interest (ROI) in the femur bone marrow (red boxes in the unprocessed reconstructions in Fig. 5). To this end, we estimate the signal strength as the average voxel brightness in the ROI, and the noise as the average voxel brightness in the background (white boxes). Prior to each acquisition, we measured the spectral noise density as picked up by the detection RF coil with the subject inside the scanner. The average signal strength in these spectra speaks of the white noise amplitude in the Rx chain, which is ideally close to thermal (Johnson) noise in the coil (Methods). Besides, we often encounter stronger peaks, indicating EMI at discrete frequencies. These can be suppressed by covering the subject meticulously with the shielding cloth.

The first acquisition (Fig. 5a) took place in the same laboratory as above and serves as reference under controlled ambient conditions. For the acquisition in the MRI physics laboratory, the Larmor frequency was $\approx 3.076$ MHz, the measured noise level ($\approx 50$ nV/Hz$^{1/2}$) was compatible with Johnson noise (Methods), there is no visible EMI and the femur SNR is $\approx 21$.

The second scan took place in an office room (Fig. 5b) around 20 m away from the laboratory, in the same building and floor. The Larmor frequency here decreased to $\approx 3.064$ MHz due to a higher temperature. The noise amplitude is still consistent with Johnson noise levels and EMI is not visible in the reconstruction. The SNR in the marrow ROI is $\approx 25$ and the overall image quality is comparable to the reference image, perhaps even slightly sharper.

The third image was acquired outdoors, at basement level, just outside the laboratory building (Fig. 5c). The system was powered through a 30 m cable running down three floors from the laboratory. The conducting cloth wrapped around the subject purposely connected the scanner shielding to the concrete floor to improve the otherwise resistive connection between the laboratory ground and earth. During this acquisition the volunteer reported sensing the presence and conversations of bypassers, a light breeze on the grounded cloth, and weak tremors due to vehicles driving through the underground parking. The resulting image quality seems not to be strongly influenced by any of these, with an SNR of $\approx 19$ in the ROI, and a noise spectrum of comparable amplitude to indoor acquisitions. The Larmor frequency was $\approx 3.065$ MHz.

The fourth scan was also taken outdoors, in this case in open air in a university campus (Fig. 5d, Larmor frequency $\approx 3.063$ MHz), far from power outlets and operating autonomously with a portable electricity generator. The latter is based on a low-consumption gasoline engine, weighs $<20$ kg, costs $<600$ € and has an autonomy $>10$ hours with the scanner at continuous operation (Methods). We grounded the system electrically as before, with the conducting cloth offering low-resistance paths between the scanner shielding, the floor concrete and the ground terminal in the generator. The spectrum was significantly more populated in this case, with a mean amplitude roughly twice the expected Johnson limit, presumably due to noise originating at the engine. Consequently, the quality of the resulting image is lower than in the previous acquisitions ($\text {SNR}\approx 11$), and an EMI line is visible along the vertical (phase-encoding) direction. Nevertheless, the main anatomic features, different tissues and metallic implants are all still clearly identifiable.

The last image was acquired at the volunteer’s apartment. This is located in a low-density town in the province of Valencia, Spain. The system was transported in a small truck from the university campus to a parking lot approximately 300 m away from the entrance to the apartment block, and pushed along the sidewalk into the building, the elevator, the apartment and ultimately, the living room. Throughout the way, the only wheelchair-adapted elements were the lowered-sidewalks at pedestrian crossings. After transport, the system required re-connecting some of the RF electronics modules we had packed in a separate box, and tightening some screwed connectors that had become loose during transport through the rugged, tiled sidewalks. Other than that, the system was plugged to a wall power outlet, tuned to $\approx 3.065$ MHz and ready to use. The noise spectrum in the apartment was clean, again compatible with Johnson noise levels. The SNR in the ROI for this acquisition is $\approx 19$.

## Discussion

In conclusion, we have demonstrated the viability of a portable, low-cost system for magnetic resonance imaging indoors, outdoors and at home. In this work, we have focused on healthy volunteers and subjects carrying metallic implants. Nevertheless, the acquired images contain sufficient anatomical information to diagnose a large variety of articular diseases, including effusion, synovial engorgement, tendon disruption or bone fractures.

System portability is a major goal for low-field systems, since this is not expected to be possible with high-field scanners in the near future. Our setup makes use of a Halbach magnet, as do others^12,29,30^. An important advantage of Halbach configurations is their reduced weight compared to yoked magnets. For instance, the 64 mT system from Hyperfine Inc. weighs $>600$ kg and the 55 mT system from Liu et al. around 750 kg^31^. In contrast, the weight of our system is comparable to that of a hospital bed ($\approx 140$ kg) with a patient ($\approx 80$ kg), making it amenable to transport by a single person on a flat surface. Therefore, even if the open design of yoked magnets eases patient handling and comfort (especially for neuroimaging), a Halbach configuration is arguably advantageous in terms of portability. Gradient efficiency is also improved in Halbach configurations in the sense that yoked magnets tend to make use of planar gradients to preserve the overall system openness. Our gradients are on cylindrical surfaces, which means that stronger gradients can be achieved for equivalent currents. Besides, we do not need the full power available from our gradient amplifiers, so one could consider trading efficiency for linearity, which may be useful for certain applications. Regarding the RF circuitry, the antennas employed in other low-field scanners are mostly dedicated head coils for neuroimaging applications. We have not yet explored this, because our scanner is somewhat small for head imaging. Finally, to complete this comparison with other existing low-field systems, we must stress that Hyperfine Inc. is well ahead of any other initiative, including ours, both in terms of having designed a final product and having certified it for clinical use. Nevertheless, future scanners with greatly enhanced portability will probably require the aforementioned benefits of Halbach magnets.

Looking ahead, our 72 mT scanner can be still upgraded in various ways. Machine learning algorithms have been shown to boost the performance in other low-field systems and can be readily incorporated to ours. These can be used, via transfer learning, to increase the spatial resolution of scans a posteriori based on multiple acquisitions, prior knowledge about the sample^32^, or with networks trained with paired datasets of low and high-field images, to recover from the former features visible otherwise only with the latter^21,33^. Deep learning and convolutional neural networks can also be employed to increase reconstruction quality through image denoising, artifact detection and active noise cancellation^20,31,34^. Quantitative MRI, radiomics and fingerprinting^16,17,35^ show promising potential in situations where subtle texture changes contain potentially valuable information for the patients. Also special-purpose pulse sequences and reconstruction methods can enhance the efficiency of low-field MRI^13,17^, and hardware developments and contrast agents which are mainstays in clinical high-field MRI (e.g. parallel imaging, optimization of RF detection coils for different body parts, gadolinium contrast enhancement), are seldom used in the still mostly experimental low-field systems available^2,36^. Finally, for our particular scanner, the GUI and overall system usability can be improved to facilitate operation by non-experts.

All in all, the scanner performance demonstrated in this work, especially if upgraded with the above capabilities, sets a path towards accessible MRI, democratizing its use and benefits, and qualitatively expanding the circumstances where it can provide value.

## Methods

### Scanner

The scanner employs a Halbach cylinder magnet including almost 4600 N48 NdFeB cubes of side 12 mm to generate $B_0\approx 72$ mT at the field of view, and another $\approx 1100$ N42 smaller cuboids (64 mm$^3$) to shim the inhomogeneity from $\approx$ 15,700 down to $\approx 3100$ ppm over a spherical volume of 20 cm in diameter. This was designed for an inner diameter of $\approx 27$ cm following methods described in Ref. ^37^, but we optimized for three magnet layers (rather than two) to increase the field strength from 50 to 72 mT. All in all, the magnet includes 23 rings held in place by 8 external screws traversing the complete setup. The Larmor frequency dependence on air temperature is $\approx -6$ kHz/C.

The gradient coil geometry is optimized with conventional target-field methods, following the procedures described in Ref. ^37^. Our *x* (axial), *y* (vertical) and *z* (horizontal) gradients have, respectively: efficiencies $\approx 0.53$, 0.91 and 0.89 mT/m/A; resistances $\approx 0.35$, 0.38 and ${0.40}\,{\Omega }$ with a wire of diameter 1.5 mm; inductances $\approx 180$, 227 and ${224}\, \upmu \hbox {H}$; and deviations $\approx 27.2$, 1.1 and 1.0 % from perfect linearity over a 15 cm DSV. These coils are wound on and glued to curved 3D-printed Nylon molds, and the whole assembly is supported by a methacrylate cylinder. We have not encountered the need for water or air cooling. The gradient analog waveforms are generated with an OCRA1 board^38^, connected to a Red Pitaya Stemlab 122.88-16 SDR^39^ via Serial Peripheral Interface (SPI), and amplified by AE Techron 7224 power amplifiers (Indiana, USA), which can deliver up to $\approx 45$ A onto our loads at relevant duty cycles. This corresponds to gradient fields of up to 25 mT/m along *x* and 40 mT/m along the *y* and *z*. Under normal operating conditions, the Larmor frequency decreases by $\approx 10$ Hz/min due to heating of the permanent magnets from the power dissipated by the gradient coils.

We used two Tx/Rx RF antennas, one for the images in Figs. 2 and 3 (of inner diameter $\approx 14$ cm), and a larger one for the implanted knee ($\approx 20$ cm). Both are solenoid coils tuned and impedance-matched to the proton Larmor frequency ($\approx 3$ MHz). The RF coil holders were 3D-printed in polylactic acid (PLA), and the wire was fixed with cyanoacrylate adhesive. The coils are inside a grounded copper shield to mitigate noise pick-up and prevent interference between the gradients and the RF system, and a conductive cloth covers the subject during in vivo acquisitions. The RF low-noise (45 dB gain, 50ohm, noise figure $<1$ dB) and power amplifiers (250 W, maximum duty cycle of 10 % with 10 ms pulses), as well as the passive Tx/Rx switch, were purchased from Barthel HF-Technik GmbH (Aachen, Germany).

The control electronics are based on MaRCoS, an open-source, high-performance Magnetic Resonance Control System^40–42^.

The diameter and length of the scanner are around 53 and 51 cm respectively, excluding electronics and the mobile structure, with a bore opening $\approx 24$ cm (inner diameter of gradient structure) and a weight of $\approx 200$ kg. Once on the mobile, open structure and equipped with all the required electronics and the control computer, the overall system dimensions are $70\times 88\times 166$ cm$^3$ and the weight is $\approx 250$ kg.

### Pulse sequences

The protocols for experiments involving human subjects were approved by the Ethics Committee (CEIm) of La Fe Hospital in Valencia (IIS-F-PG-22-02, research agreement number 2019-139-1).

Some aspects common to all the images presented in this work are: (i) the duration of resonant $\pi /2$ and $\pi$-pulses in all images are $\approx {40}\, \upmu \hbox {s}$ and $\approx \, {80}\upmu \hbox {s}$, respectively; (ii) the readout dephasing gradient pulses after the RF excitation pulses are pre-emphasized by a factor $\approx 1.008$ to place the echoes at the center of the data acquisition windows and mitigate the effects of imperfect gradient waveforms and induced Eddy currents; and (iii) an automatic Larmor-frequency calibration is run before every new scan, i.e. full sequence for an image.

The knee image in Fig. 2a was acquired with a $T_1$-weighted 3D-RARE sequence, with $\text {FoV} = 130\times 140\times 180$ mm$^3$, a resolution of $1.85\times 1.75\times 2$ mm$^3$, $\text {ETL} = 5$, $\text {TE} = 20$ ms, $\text {TR} = 200$ ms, $\text {BW} = 17.5$ kHz, and 4 averages for a total scan time of 19.2 min. The *x*, *y* and *z* axes correspond to the readout (RO), phase-encoding (PE) and slice-encoding (SE) directions, respectively.

The hand image in Fig. 2b was acquired with a $T_1$-weighted 3D-RARE sequence, with $\text {FoV} = 180\times 180\times 50$ mm$^3$, a resolution of $1.5\times 1.5\times 5$ mm$^3$, $\text {ETL} = 10$, $\text {TE} = 20$ ms, $\text {TR} = 400$ ms, $\text {BW} = 30$ kHz, and 13 averages for a total scan time of 10.4 min. The *x*, *y* and *z* axes correspond to the RO, SE and PE directions, respectively.

The wrist image in Fig. 2c was acquired with a $T_1$-weighted 3D-RARE sequence, with $\text {FoV} = 180\times 140\times 80$ mm$^3$, a resolution of $1.5\times 1.5\times 10$ mm$^3$, $\text {ETL} = 3$, $\text {TE} = 20$ ms, $\text {TR} = 100$ ms, $\text {BW} = 30$ kHz, and 30 averages for a total scan time of 12 min. The *x*, *y* and *z* axes correspond to the RO, SE and PE directions, respectively.

The wrist image in Fig. 2d was acquired with a $\rho$-weighted 3D-RARE sequence, with $\text {FoV} = 180\times 140\times 80$ mm$^3$, a resolution of $1.5\times 1.5\times 10$ mm$^3$, $\text {ETL} = 5$, $\text {TE} = 20$ ms, $\text {TR} = 1000$ ms, $\text {BW} = 30$ kHz, and 5 averages for a total scan time of 12 min. The *x*, *y* and *z* axes correspond to the RO, SE and PE directions, respectively.

The wrist image in Fig. 2e was acquired with a $T_2$-weighted 3D-RARE sequence, with $\text {FoV} = 180\times 140\times 80$ mm$^3$, a resolution of $1.5\times 1.5\times 10$ mm$^3$, $\text {ETL} = 5$, echo spacing of 20 ms, effective $\text {TE} = 100$ ms, $\text {TR} = 1000$ ms, $\text {BW} = 30$ kHz, and 5 averages for a total scan time of 12 min. The *x*, *y* and *z* axes correspond to the RO, SE and PE directions, respectively.

The knee images in Fig. 3 were acquired with a $T_1$-weighted 3D-RARE sequence, with $\text {FoV} = 150\times 150\times 180$ mm$^3$, a resolution of $1.50\times 1.85\times 10$ mm$^3$, $\text {ETL} = 5$, $\text {TE} = 20$ ms, $\text {TR} = 200$ ms, $\text {BW} = 25$ kHz, and 12 averages for a total scan time of 11.5 min. The *x*, *y* and *z* axes correspond to the SE, PE and RO directions, respectively.

The knee images in Fig. 4a,b were acquired with a $T_1$-weighted 3D-RARE sequence, with $\text {FoV} = 200\times 200\times 180$ mm$^3$, a resolution of $1.3\times 2\times 9$ mm$^3$, $\text {ETL} = 5$, $\text {TE} = 20$ ms, $\text {TR} = 200$ ms, $\text {BW} = 37.5$ kHz, and 9 averages for a total scan time of 12 min. The *x*, *y* and *z* axes correspond to the RO, PE and SE directions, respectively.

The knee image in Fig. 4e was acquired with a $T_1$-weighted 3D-RARE sequence, with $\text {FoV} = 200\times 200\times 180$ mm$^3$, a resolution of $2\times 2\times 2$ mm$^3$, $\text {ETL} = 10$, $\text {TE} = 20$ ms, $\text {TR} = 300$ ms, $\text {BW} = 22.5$ kHz, and 4 averages for a total scan time of 20 min. The *x*, *y* and *z* axes correspond to the RO, PE and SE directions, respectively.

The knee images in Fig. 5 were acquired with a $T_1$-weighted 3D-RARE sequence, with $\text {FoV} = 180\times 200\times 200$ mm$^3$, a resolution of $1.2\times 2\times 10$ mm$^3$, $\text {ETL} = 5$, $\text {TE} = 20$ ms, $\text {TR} = 200$ ms, $\text {BW} = 37.5$ kHz, and 9 averages for a total scan time of 12 min. The *x*, *y* and *z* axes correspond to the SE, PE and RO directions, respectively.

### Data acquisition

The receive chain consists of an analog stage (RF coil, passive Tx/Rx switch and low-noise amplifier) followed by a digital stage. The digitization is performed at 122.88 Ms/s by an analog-to-digital converter in the Red Pitaya Stemlab board^40–42^. The digital signal is mixed down by complex multiplication with a numerically-controlled oscillator set to the Larmor frequency. The real and imaginary data components pass first a cascaded integrator-comb filter and finally a finite impulse response filter. The resulting data conform the sought in-phase and quadrature components of the magnetic resonance signal. These are sent to the control computer and can be Fourier-transformed for image reconstruction and post-processing.

### Image reconstruction and post-processing

All images have been reconstructed directly by an Inverse Fast Fourier Transform protocol implemented in the MaRCoS GUI we have developed in Python^40^. The presented reconstructions are therefore subject to distortions due to field inhomogeneity and gradient non-linearities. These can be mitigated by reconstruction algorithms which include information on the field maps^43^, but we have not found this necessary at this stage. The only post-processing operations we have used in this work are BM4D-filtering^27^ and image rescaling to increase the number of pixels, and only where explicitly indicated in the main text.

### Noise

The spectral noise density of the MR data is bounded from below by Johnson noise due to thermal fluctuations of electrons in the resistive elements *R* in the receive chain (up to the LNA). These are dominated by the coil, with quality factor $Q\approx 93$ (88) and $R\approx 5$ $({5.5}\,{\Omega })$ in the unloaded (loaded) case. For a given acquisition bandwidth, the integrated noise amplitude is expected to be $(4k_\text {B} R\cdot BW)^{1/2}$, with $k_\text {B}$ the Boltzmann constant. In the controlled environment of the MRI physics laboratory, we measure $\approx 50$ nV/Hz$^{1/2}$ after a 45 dB low-noise pre-amplifier, in agreement with the estimated Johnson level. We use this as a reference to evaluate the signal quality and the shielding efficiency of the conductive cloth, both in the laboratory and in the rest of locations.

We have found situations where suppressing noise down to Johnson levels is not trivial, and indeed did not achieve it when the system was powered by the portable generator. The control computer is another significant source of 50 Hz noise and needs to be as far as possible in the rack to reconstruct clean images. We also find it often necessary to ensure the subject is sufficiently covered by the conductive cloth, and extending some of it on the floor helps.

### Generator

For the autonomous experiments outdoors we powered the system from a “Limited 2000i” gasoline-fueled generator from Genergy (Calahorra, Spain). This motor delivers up to 2 kW at 230 V and 50 Hz (single phase). It costs $<600$ €, weighs 19 kg and has a fuel tank capacity of 4 l and an autonomy of 10.8 hours at 25 % load (500 W), which is more than required for continuous operation of the scanner.

### Ethical approval

All experiments were carried out in accordance with the guidelines in the Declarations of Helsinki and following Spanish regulations and under the research agreement from La Fe Hospital in Valencia (IIS-F-PG-22-02, agreement number 2019-139-1).

### Consent to participate

Written informed consent was obtained from all subjects prior to study commencement.

### Consent for publication

Written informed consent for publication was obtained from all subjects prior to study commencement.

## Data Availability

All anonymized datasets, reconstruction and post-processing methods generated and/or used during the present study are available from the corresponding author upon reasonable request.

## Code availability

The codes for MaRCoS and the GUI are publicly available from open-source repositories at https://github.com/vnegnev/marcos_server and https://github.com/yvives/PhysioMRI_GUI, respectively.
